# Boosting photocatalytic water splitting of TiO_2_ using metal (Ru, Co, or Ni) co-catalysts for hydrogen generation

**DOI:** 10.1038/s41598-024-59608-0

**Published:** 2024-05-02

**Authors:** Safinaz M. Thabet, Hani Nasser Abdelhamid, Said A. Ibrahim, Haitham M. El-Bery

**Affiliations:** 1 Department of Chemistry, Faculty of Science, Assiut University, Assiut, 71515 Egypt; 2 Egyptian Russian University, Badr City, 11829 Cairo Egypt; 3 Basics Science Department, School of Biotechnology, Badr University in Assiut, Assiut, 2014101 Egypt

**Keywords:** Photocatalytic, Water splitting, Hydrogen production, Photocatalytic deposition, Impregnation, Hydrothermal, Environmental sciences, Energy science and technology, Materials science

## Abstract

The photocatalytic activity of titanium dioxide (TiO_2_) nanoparticles toward hydrogen generation can be significantly improved via the loading of various metals e.g., Ru, Co, Ni as co-catalysts. The metal co-catalysts are loaded into TiO_2_ nanoparticles via different deposition methods; incipient wet impregnation (Imp), hydrothermal (HT), or photocatalytic deposition (PCD). Among all of the tested materials, 0.1 wt% Ru–TiO_2_ (Imp) provided the highest initial hydrogen catalytic rate of 23.9 mmol h^−1^ g^−1^, compared to 10.82 and 16.55 mmol h^−1^ g^−1^ for 0.3 wt% Ni–TiO_2_ (Imp) and 0.3 wt% Co–TiO_2_ (Imp), respectively. The loading procedures, co-catalyst metals type, and their loading play a significant role in elevating the photocatalytic activity of pristine TiO_2_ semiconductors toward hydrogen generation. Redox transition metals e.g., Co and Ni exhibit comparable photocatalytic performance to expensive elements such as Ru.

## Introduction

Hydrogen (H_2_) is a green source of energy with no emission of greenhouse gases offering high energy density (ca., 120 MJ/kg) and lower volumetric energy density (ca., 8 MJ/L)^1–6^. It can help to mitigate climate change due to greenhouse gases released from the combustion of fossil fuels^7,8^. It can be generated via several methods including water electrolysis^7,9,10^, gas reformation^11,12^, hydrolysis of hydrides^13–17^, and biomass gasfication^18^. Each of these methods exhibits pros and cons^19–22^. Overall, they can provide hydrogen with high purity, and techno-economical reasonable costs, and can be used for large production. However, most of these methods require non-renewable sources^3^. There are several challenges in hydrogen production including high cost, transportation/storage, and insufficient infrastructure^23,24^. The method for hydrogen production should be environmentally friendly during generation and use a renewable source with minimal energy requirements.

Photocatalytic water splitting is a promising method for hydrogen generation^25,26^. The topic was reviewed for titanium dioxide (TiO_2_) semiconductors in several reviews including Ref.^27–31^. The photocatalytic efficiency of TiO_2_ is low due to several challenges such as fast charge recombination of the photogenerated species. The optimization of key parameters such as co-catalyst types and loading percentage or procedure may improve the photocatalytic performance for TiO_2_^32–34^. A study comparing different preparation methods of heavy metal co-catalysts for hydrogen generation is rare^35,36^.

Considering the previous points, this study shed light on the influence of type, loading percentage, and deposition method of metals (e.g., Ru, Co, and Ni) as co-catalysts on the photocatalytic performance of TiO_2_ for water splitting. Three metal types were investigated including ruthenium (Ru), nickel (Ni), and cobalt (Co). The metal ions were loaded into TiO_2_ using a loading percentage of 0.05–1 wt.% in the case of Ru and 0.1–1 wt.% in the case of Co and Ni. The loading procedure was investigated using three different procedures namely; impregnation (Imp), photocatalytic deposition (PCD), and hydrothermal (HT). X-ray diffraction (XRD), X-ray photoelectron spectroscopy (XPS), transmission electron microscopy (TEM), high-resolution TEM (HR-TEM), and UV–Vis diffuse reflectance spectroscopy (DRS) were used to characterize the materials. Under UV light irradiation, the hydrogen generation tests were carried out in a closed flow system with argon gas as the carrier gas and methanol serving as the sacrificial electron donor. There are high hydrogen generation rates (HGRs) in the photocatalysts. Electrochemical tests such as cyclic voltammetry (CV) and electrochemical impedance spectroscopy (EIS) were used to investigate the improvement's mechanism.

## Experimental

### Chemicals and methods

TiO_2_ was obtained from Acros Organics. Ethanol (99%), methanol (99%), isopropanol (99%), and metal precursors (RuCl_3_**·**2H_2_O, Ni(NO_3_)_2_**·**6H_2_O, Co(NO_3_)_2_**·**6H_2_O) were obtained from Fisher Scientific (UK, LTD).

### TiO_2_-based nanocomposite preparation

Different loading percentages of metal e.g., Ru, Co, and Ni were loaded into TiO_2_ nanoparticles via three different methods; Imp, HT, and PCD respectively. The obtained powder will be denoted as xM/TiO_2__y; where x, M, and y represent the weight percent, the metal loading, and the deposition method of the metal co-catalysts on TiO_2_, respectively. The three procedures can be described below:-

### Incipient wet impregnation method (Imp)

TiO_2_ was suspended in 3 mL using an alumina crucible. After that, the mixture was stirred by a glass rod and ultrasonicated. The suspension was powdered by placing the crucible over a hot water bath with constant stirring till complete dryness. The powder sample was then moved to a tube furnace and purged by Ar (99.99%) using a flow rate of 100 mL min^−1^ for 15 min. After the purge, H_2_ (99.99%) gas flowed at a rate of 10 mL min^−1^, and the furnace temperature was raised to 200 °C (at a rate of 10 °C min^-1^) and held for 1 h. The powder was cooled naturally under the flow of H_2_ gas.

### Hydrothermal method (HT)

A suspension of TiO_2_ powder (1 g) was prepared in 90 mL of distilled water and then sonicated for 10 min. For loading the required ratio of metal to TiO_2_, the studied volume of the metal precursor was mixed with 5 mL ethanol and then added dropwise to the TiO_2_ suspension, the reaction was maintained under vigorous stirring for one hour. The dispersion was heated at 180 °C for 12 h using a Teflon-lined stainless steel autoclave. The materials were collected and washed with water.

### Photocatalytic deposition (PCD)

In the photo-deposition method, the reduction can be achieved via the photon energy-assisted methanol method. Briefly, the metal precursor was added to 200 mL of 20% methanol aqueous suspension containing TiO_2_ and then transferred into a Pyrex cell. After the purge with Ar gas, the cell was subjected to a UV-LED lamp for three hours. The precipitate was collected, washed using distilled H_2_O, and dried.

### Photocatalytic H_2_ production experiments

The photocatalytic hydrogen generation was measured via an online system. As shown in Fig. S1, a Pyrex glass reactor connected to a flow system was set for photocatalytic evaluation of hydrogen gas. Typically, 50 mg of the photocatalyst was suspended in the reactor containing 200 mL of 20 vol.% methanol aqueous solution as a hole scavenger and subjected to constant stirring (800 rpm). Before irradiation, the suspension was ultrasonicated for 5 min and purged for 30 min with Ar gas. After that, the Ar gas flow rate was decreased to 10 mL min^−1^, and the suspension cell was situated at a 1 cm distance from the illumination UV–LED light source (25 W, 365 nm, NVMUR020A, NICHIA, Japan). The amount of gas evaluated was detected every 15 min using a gas-chromatography (GC) system (Shimadzu, GC-2014, Shin Carbon ST 80/100 with Length: 2 m and ID:2 mmID column, thermal conductivity detector, and argon gas as the carrier). The experiment lasted for 300 min at room temperature.

Photoelectrochemical measurements were performed using a potentiostat workstation (CorrTest^®^ Instruments, model CS350) an electrolyte of Na_2_SO_4_ aqueous solution (40 mL, 0.1 M). The working electrode was prepared by casting the materials into FTO substrates with an active area of ca. 1.0 cm^2^. The counter and reference electrodes were a Pt wire and Ag/AgCl, respectively. Cyclic voltammetry (CV) and electrochemical impedance spectroscopy (EIS) measurements were recorded with and without light.

The working electrodes were prepared using the electrophoretic deposition method. Typically, the photocatalyst (20 mg) was dispersed in 1 mL isopropanol and sonicated for 15 min to make a homogenous slurry or paste. 50 µL of the slurry was deposited into an FTO conducting glass substrate that was then dried in air. The materials were deposited via layer-by-layer procedures. Finally, the electrodes were dried in an oven and calcinated at 120 °C for 60 min.

### Characterization instruments

XRD patterns were collected using Bruker D8 advance equipment (Cu K_α_ radiation). XPS spectra were recorded using Al K_α_ radiation (Thermo Scientific, USA). TEM and HR-TEM images were collected using JSM-2100 (JEOL, Japan). The ultraviolet–visible (UV–Vis) DRS of powder samples were collected using an Evolution 220 spectrophotometer (Thermo Fisher Scientific, UK). Tauc’s plots were used to determine the materials band gap. Nitrogen adsorption–desorption isotherms were determined using Quantachrome (USA, at 77 K). Pore size distribution (PSD) was determined using the Barrett-Joyner-Halenda (BJH) method.

## Results and discussion

### Photocatalysts synthesis and characterization

**Figure 1:**
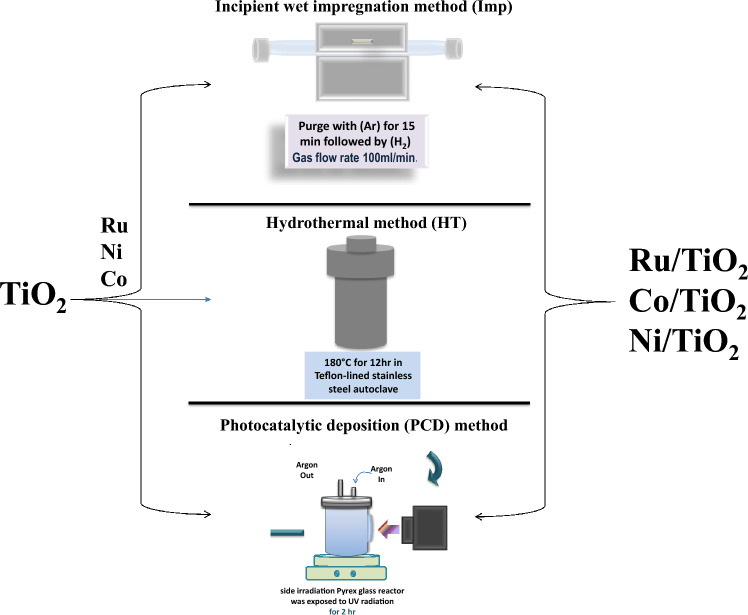
Schematic representation for Imp, HT, and PCD methods used for loading Ru, Co, and Ni into TiO_2_.

**Figure 2:**
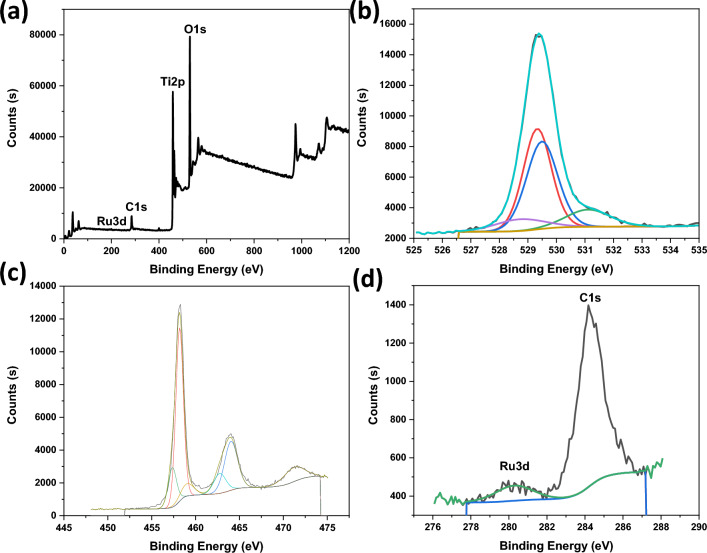
XPS for Ru/TiO_2_, (**a**) survey, (**b**) O1s, (**c**) Ti2p, and (**d**) Ru3d.

**Figure 3:**
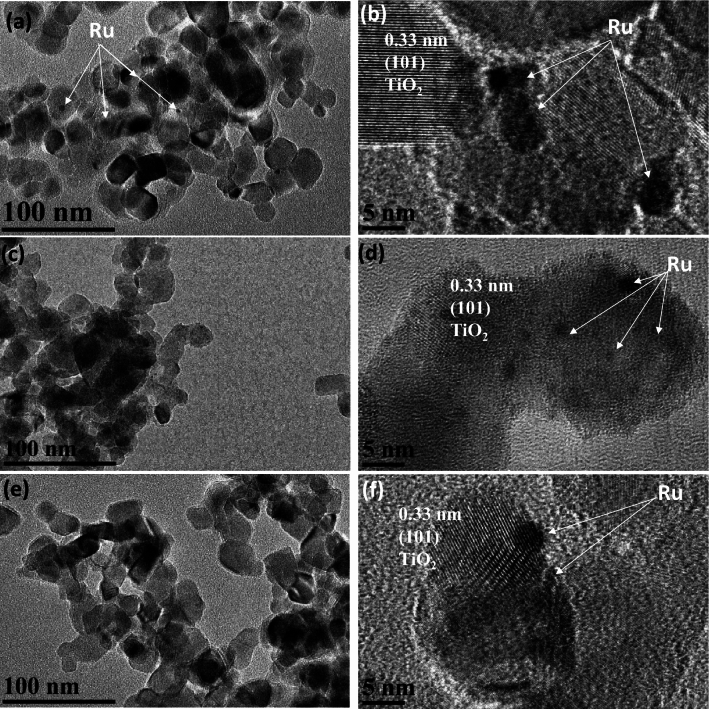
TEM and HR-TEM images for Ru/TiO_2_ for loading methods of (**a**,**b**) HT), (**c**,**d**) Imp and (**e**,**f**) PCD.

**Figure 4:**
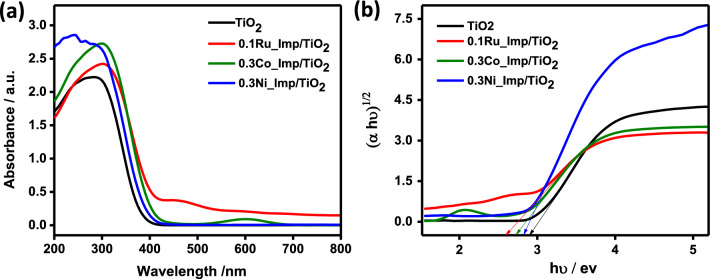
(**a**) DRS spectra and (**b**) the band gap energies for 0.1 wt.%Ru_TiO_2_ (Imp), 0.3 wt.%Co_TiO_2_ (Imp), and 0.3 wt.%Ni_TiO_2_ (Imp) loaded into TiO_2_.

Three different methods; namely Imp (Fig. S2), HT (Fig. S3), and PCD (Fig. S4), were used for the synthesis of metal-loaded TiO_2_ (Fig. 1). Metal ions of Ru^3+^, Co^2+^, and Ni^2+^ ions were used for loading offering composites of Ru/TiO_2_, Co/TiO_2_, and Ni/TiO_2_, respectively. The Imp method involves metal ions adsorption into the TiO_2_ substrate via sonication and stirring (Fig. S2). The metal ions were then reduced via hydrogen gas at 200 °C (Fig. S2). HT method includes the same procedure of loading, except that the metals loaded TiO_2_ were then subjected to hydrothermal treatment at 280 °C for 2 h (Fig. S3). Following the same loading procedure, PCD was performed (Fig. S4). PCD involves the reduction of the metal loading using a light source (Fig. S4). All procedures offer the synthesis of Ru/TiO_2_, Ni/TiO_2_, and Co/TiO_2_. The materials are fully characterized using XRD (Fig. S5), XPS (Fig. 2, Figs. S6, S7), TEM/HR-TEM (Fig. 3), N_2_ adsorption–desorption isotherms (Fig. S8), pore size distribution (PSD, Fig. S9), and UV–Vis DRS (Fig. 4, Figs. S10, S11). These techniques characterize the material's crystallinity, phase, oxidation state, particle size, morphology, porosity, pore size distribution, and optical properties including bandgap.

XRD patterns for the materials were recorded to confirm the crystallinity and phase purities (Fig. S5). They display characteristic diffraction peaks at Bragg angles (2θ) of 25.2°, 36.1°, 48.0°, 53.9°, and 55.0° corresponding to the Miller indexes of (101), (004), (200), (105), and (211), respectively for anatase TiO_2_ (JCPDS No. 21-1271). The other peaks at Bragg angles of 27.4°, 36.1°, 41.2°, 54.3°, 56.6°, 62.7°, and 69.0° were assigned to (110), (101), (111), (211), (220), (002) and (301), respectively for Rutile TiO_2_ (JCPDS No. 21-1276). The TiO_2_ is a homophase junction of rutile and anatase phases. There is no strong diffraction of the loaded metals indicating that these species are amorphous or their loadings are undetectable in the XRD patterns. Thus, XPS for Ru/TiO_2_ was recorded (Fig. 2). XPS survey confirms the presence of Ti, Ru, and O at binding energies 458.3 eV, 280.2 eV, and 529.9 eV indicating the formation of Ru/TiO_2_ composite (Fig. 2a). Data analysis for Ru3d shows the peak at 280.27 eV corresponding to Ru^0^ indicating that the Imp method offers the synthesis of metallic Ru (Fig. 2d). XPS analysis was also performed for Co/TiO_2_ and Ni/TiO_2_ prepared by the Imp method (Figs. S6, S7). The Co2p_3/2_ peak in the XPS analysis at 780.9 eV corresponds to Co^2+^ in CoO (Fig. S6d). While XPS for Ni2p_3/2_ shows a peak at 857.2 eV corresponding to Ni^2+^ in NiO (Fig. S7d).

TEM and HR-TEM images for Ru/TiO_2_ were recorded for loading methods of Imp, HT, and PCD (Fig. 3). Two different particles are observed in TEM images corresponding to TiO_2_ and Ru (Fig. 3). Irregular particles of TiO_2_ with a particle size of 10–50 nm (Fig. 3). The particles of TiO_2_ can be confirmed from the lattice fringes observed using HR-TEM images (Fig. 3). TiO_2_ displays lattice fringes of 0.33 nm (101). The high electron density in Ru causes the observation of dark particles located in the plane of (101) for TiO_2_ (Fig. 3). N_2_ adsorption–desorption isotherms and PSD of the materials were recorded as shown in Figs. S8 and S9, respectively. Data analysis shows BET-specific surface areas of 70–203 m^2^/g with a pore size of 1.5–2 nm (Figs. S8, S9). It is important to mention that most of these porosities refer to the interparticle pores formed between metal-loaded and TiO_2_ crystals i.e., interparticle porosity according to TEM images (Fig. 4).

The optical absorption of the materials was determined using DRS (Fig. 4, Figs. S10, S11). The band gap values were determined using Tauc’s plots. UV–Vis spectra of all materials exhibit the characteristic absorption peak for TiO_2_ at maximum absorption at wavelength 300 nm (Fig. 4, Figs. S10, S11). TiO_2_ shows a bandgap of 2.97 eV (Fig. 4b). Transition elements cause a decrease in the bandgap values to a lower value of 2.6–2.8 eV. The drop in the bandgap of TiO_2_ indicates the formation of heterojunction between the transition elements and TiO_2_ nanoparticles. This effect can tune the material’s photocatalytic performance.

### Photocatalytic water splitting

**Figure 5:**
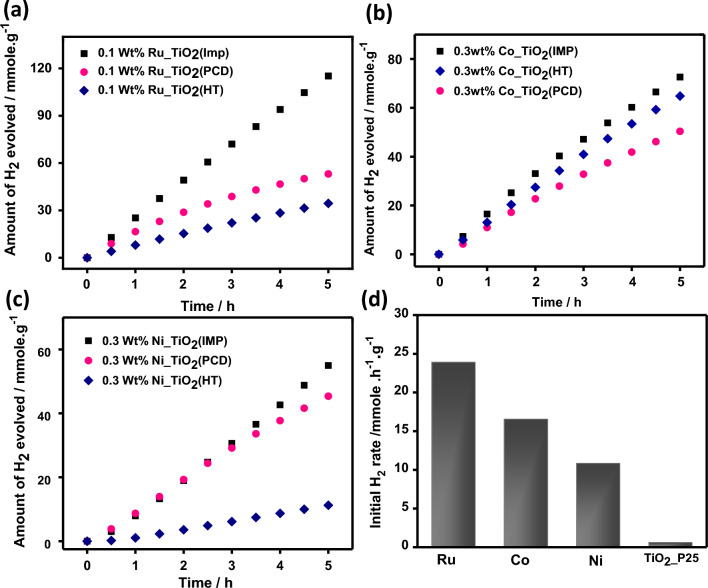
Time course of H_2_ evolution over 50 mg samples of (**a**) 0.1 wt.%Ru_TiO_2_, (**b**) 0.3 wt.%Co_TiO_2_ and (**c**) 0.3 wt.%Ni_TiO_2_ photocatalysts with different metal loading methods, (**d**) shows the relation between initial H_2_ production rates over 50 mg of 0.1 wt.%Ru_TiO_2_ (Imp), 0.3 wt.%Co_TiO_2_ (Imp), and 0.3 wt.%Ni_TiO_2_ (Imp) nanocomposites relative to TiO_2_ in 20 vol% methanol aqueous solution subjected to UV_light for 5 h.

The photocatalytic activity of TiO_2_-based composite is tested. The effects of metal types (e.g. Ru, Co, and Ni), loading procedures (Fig. 5), and loading percentage (Fig. S12) were investigated. The HGR can be arranged in the sequence of Ru > Co > Ni (Fig. 5). HGR values for Ru/TiO_2_, Co/TiO_2_, and Ni/TiO_2_ were 23.91, 16.55, and 10.83 mmol/g h, respectively. Electron-rich elements such as Ru exhibit high HGR.

The initial and cumulative hydrogen rate was observed for 0.1, 0.3, and 0.3 wt.% of Ru, Ni, and Co metals, respectively. High loading of these metals causes a decrease in the initial and cumulative rates. These observations could be due to the light block caused by high loading that prevents light radiation from reaching the external surface of TiO_2_ semiconductors.

**Figure 6:**
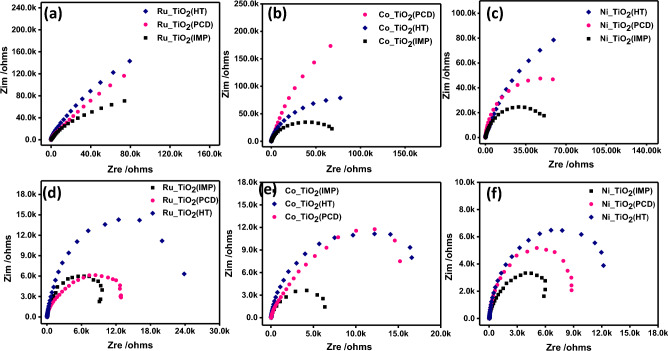
(**a**–**c**) Nyquist plots of the EIS data in dark conditions for Ru_TiO_2_, Co_TiO_2_, Ni_TiO_2_ nanocatalysts of different metal loading methods and (**d**–**f**) represent Nyquist plots of the EIS data under UV light illumination in 0.1 M Na_2_SO_4_ electrolyte.

**Figure 7:**
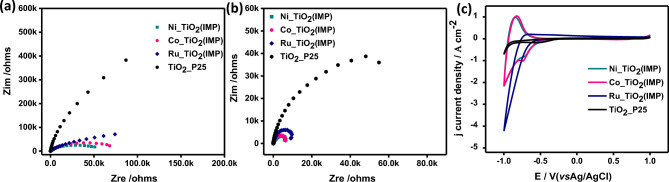
(**a**,**b**) Nyquist plots of the EIS data in (**a**) dark conditions and (**b**) under UV light illumination respectively, whereas (**c**) CV for 0.3 wt.%Ni_TiO_2_ (Imp), 0.3 wt.%Co_TiO_2_ (Imp), and 0.1 wt.%Ru_TiO_2_ (Imp) using 0.1 M Na_2_SO_4_ electrolyte.

The effect of the loading method was investigated using Imp, HT, and PCD (Fig. 5, Fig. S12). The methods of metal loading affect the distribution of the co-catalyst e.g. Ru, Ni, and Co. Thus, they affect the composite’s catalytic performance. Among the three loading methods, Imp exhibits a high catalytic performance (Fig. 5, Fig. S12). The high photocatalytic performance of the materials synthesized using Imp could be due to the homogenous distribution of the loaded metals into TiO_2_ according to TEM images (Fig. 3). The decrease of the bandgap of TiO_2_ using the Imp procedure is another explanation of this observation (Fig. 4). Further investigations were recorded using electrophotochemical measurements via EIS (Fig. 6) and CV curves (Fig. 7 and Fig. S13).

EIS spectra using the Nquists plot of all materials were recorded with and without light radiation (Fig. 6). Based on the analysis of Nquists plots, the small circle indicates low impedance i.e., high conductivity. All metal types i.e., Ru, Co, and Ni exhibit small circles for Imp compared to other loading procedures The same observation can be noticed without (Fig. 6a–c) and with light radiation (Fig. 6d–f). Furthermore, the size of all circles is smaller under UV radiation compared to without light.

Based on EIS analysis using Nyquist plots (Fig. 7), the co-catalysts of Ru, Ni, and Co exhibit small circles compared to bare TiO_2_. The presence of these cocatalysts increases the conductivity of the semiconductor TiO_2_. This observation were noticed without light radiation (Fig. 7a) and with light radiation (Fig. 7b). There is a dramatic decrease in the circle size indicating high conductivity for the composites compared to the bare TiO_2_. CV curve for bare TiO_2_ and Ru_TiO_2_ exhibit only cathodic reduction profile (Fig. 7c). On the other side, Co_TiO_2_ and Ni_TiO_2_ exhibit redox properties i.e. reduction–oxidation peaks. All prepared photocatalysts by the Imp method showed higher cathodic current than the others prepared by HT and PCD methods (Fig. S13).

The mechanism of H_2_ generation via water splitting requires a photocatalyst with a negative conduction band potential higher than the values for water reduction potential (− 0.41 eV). The photocatalyst should also display a positive valence band value than the water oxidation potential i.e., 1.23 eV. TiO_2_ semiconductor displays suitable band gap for water splitting. However, the high rate for the recommendation of the created electron–hole. Thus, bare TiO_2_ exhibits lower HGRs (Fig. 5). Moreover, we can improve the photocatalytic performance via the addition of co-catalysts such as Ru, Co, and Ni (Fig. 5). These co-catalysts improved also the conductivity (Figs. 6, 7) of pristine TiO_2_ enabling high electrochemical performance that can be reflected in high HGR values (Fig. 5).

A summary of some photocatalysts used for water splitting is tabulated in Table 1. Our study reported several parameters that can enhance the photocatalytic performance of well-known semiconductors i.e., TiO_2_. The co-catalysts properties such as metal types, loading, and deposition procedures play significant roles in the materials' performance. Our photocatalyst composites exhibit high HGRs compared to several reported materials (Table 1). Aqueous solutions of the chloride salts of Ru, Pd, and Ag were impregnated into TiO_2_ anatase with a content of 30 wt.% (Table 1)^37^. RuO_2_\TiO_2_ exhibited higher photocatalytic activity compared to PdO\TiO_2_ and Ag_2_O\TiO_2_^37^. Ni\Pt@TiO_2_ (110) offered high photocatalytic HGRs from methanol alcohols under ultrahigh vacuum (UHV)^38^. Ni (2.21 wt.%)/Pt/black TiO_2−x_ was synthesized via photodeposition (Table 1)^39^. The composite contains metallic Ni and Pt (i.e., 2 at.%)^39^. The material exhibited high charge separation efficiency. It showed HGRs of 69 and 3.1 mmol g^–1^ h^–1^ under UV–Vis and visible light, respectively (Table 1). The presence of Pt was essential to obtain metallic Ni instead of Ni(OH)_2_ (Table 1)^39^. The prepared photocatalysts in our study using inexpensive metals e.g., Co and Ni exhibit comparable HGRs to expensive co-catalysts such as Ru (Table 1). The high performance of these transition elements is mainly due to their high electrochemical performance. Co-catalysts such as Ni or Co nanoparticles enhanced the charge migration and separation of photogenerated species on TiO_2_^40^. These metal species i.e., Ni^2+^ improved the electron transfer inside TiO_2_ due to their low potential (Ni^2+^ + 2e^−^ = Ni, E^o^ = − 0.23 V)^41^. Thus, different forms of Ni were reported including Ni^42,43^, Cu–Ni^44^, Ni–Pd^45^, NiO^46–49^, and hydroxides (Ni(OH)_2_, Table 1)^50^. The presence of oxygen vacancy inside the composite improved the photocatalytic performance of TiO_2_. A study showed that CoO/h-TiO_2_ exhibited Z-scheme heterostructures with oxygen vacancy^51^. The authors observed that these vacancies were created during the composite formation and enhanced the photocatalytic H_2_ generation of TiO_2_ with an HGR value of 129.75 μmol h^–1^ (Table 1)^51^. However, the synthesis procedure required several steps and calcination at high temperatures. On the other side, our synthesis protocols are simple. Our catalysts exhibit high HGR values (Table 1).

**Table 1:** Summary of TiO_2_-based photocatalysts used for water splitting.

Photocatalysts	Synthesis method	Conditions	Light source	Photocatalytic conditions	Detection method	HGR	Time	Reference
RuO_2_\TiO_2_	Impregnation	Mixing Chloride salts of Ru, Pd, and Ag with TiO_2_ anatase Drying at 110 °C Calcination at 500 °C for 2 h	UV lamp (125 W)	Catalyst, 2 gL^−1^ RuO_2_/TiO_2_; 10 gL^−1^ TiO_2_ anatase, 1.5% activated carbon, pH of 1, 6% methanol, Metal loading 30 wt%	Bacharach hydrogen detector (USA), a water manometer	375 μmol	8 h	^37^
Ni/Pt/TiO_2−x_	Photodeposition	Annealing at 350 °C for 60 min in Ar Irradiated for 4 h under UV–Vis light (λ > 250 nm, 250 W)	250 W ultraviolet–visible lamp	Catalyst, 80 mg, 10 vol%methanol; water 800 mL; UV (36 Wm^−2^) -Vis light (9400 Wm^−2^)	Water displacement GC-TCD	69 and 3.1 mmol g^-1^	100 min	^39^
CoO/h-TiO_2_	Co-precipitation Calcination	Acid (HF) hydrolysis Hydrothermal, at 200 °C for 24 h Drying at 60 °C for 12 h Calcination at 600 °C for 4 h in 5% H_2_/Ar Precipitation at 180 °C for 12 h Dying at 60 °C for 12 h Calcination at 600 °C for 4 h	Xenon lamp source of 300 W, and AM 1.5G filter (365 and 420 nm irradiation light were 1.96 and 38.50 mW·cm^–2^)	Catalyst, 50 mg, 100 mL, 20 vol% methanol	GC-TCD	129.75 μmol·h^–1^	20 h	^51^
Ru_TiO_2_	Hydrothermal Impregnation Photodeposition	Hydrothermal, at 180 °C for 24 h Impregnation, RT for 10 min, 200 °C for 1 h PD, Stirring for 10 min, light radiation for 2 h	Xenon lamp source of 300 W Xenon lamp source of 300 W	Catalyst, 50 mg, 100 mL, 20 vol% methanol Catalyst, 50 mg, 100 mL, 20 vol% methanol	GC-TCD	0.1 wt% 23.9 mmol h^-1^ g^-1^	4 h	Here
Ni_TiO_2_						0.3 wt.% 10.82 mmol h^-1^ g^-1^		
Co_TiO_2_						0.3 wt.% 16.55 mmol h^-1^ g^-1^		

## Conclusions

This work presents an effective approach was reported to improve the photocatalytic performance of TiO_2_ nanoparticles for the production of hydrogen. The use of several metal co-catalysts e.g., Ru, Co, Ni applied in diverse ways (incipient wet impregnation, hydrothermal, and photo-deposition) resulted in a substantial enhancement in hydrogen generation compared to the pristine TiO_2_. Ru–TiO_2_, synthesized using the incipient wet impregnation method, exhibited the highest initial rate of hydrogen evolution (i.e., 23.9 mmol h^−1^ g^−1^), exceeding both Co and Ni counterparts. The results emphasize the crucial significance of the co-catalyst type, loading quantity, and deposition process in enhancing the photocatalytic efficiency of TiO_2_ for generating hydrogen using solar energy. Moreover, the similar performance of redox transition metals (Co and Ni) compared to the pricier Ru indicates potential opportunities for cost-efficient photocatalysts in the production of hydrogen.

### Supplementary Information

Supplementary Figures.

## Data Availability

The datasets used and/or analyzed during the current study are available from the corresponding author upon reasonable request.
