# Supplementary material for: Boosting photocatalytic water splitting of TiO2 using metal (Ru, Co, or Ni) co-catalysts for hydrogen generation
Source: Sci Rep. 2024 May 2;14:10115. doi: 10.1038/s41598-024-59608-0 (PMC11066123; doi:10.1038/s41598-024-59608-0)
Supplement: Supplementary file 1 — Supplementary Figures. [file 41598_2024_59608_MOESM1_ESM.docx]

**Boosting Photocatalytic Water Splitting of TiO_2_ using Metal (Ru, Co, or Ni) Co-catalysts for Hydrogen Generation**

Safinaz M. Thabet^1^, Hani Nasser Abdelhamid^1,2*^, Said A Ibrahim^1^, Haitham M. El-Bery^1,3^*

^1^Department of Chemistry, Faculty of Science, Assiut University, Assiut 71515, Egypt

^2^Egyptian Russian University, Badr City, Cairo, 11829, Egypt

^3^Basics Science Department, School of Biotechnology, Badr University in Assiut, Assiut, 2014101 Egypt

Corresponding Author: Abdelhamid ([hany.abdelhamid@aun.edu.eg](mailto:hany.abdelhamid@aun.edu.eg)); El-Bery ([haitham.el-bery@aun.edu.eg](mailto:haitham.el-bery@aun.edu.eg))

**Figure S1** Schematic representation for photocatalytic hydrogen generation flow system.

**Figure S2** Schematic representation for incipient wet impregnation method (IMP).

**Figure S3** Schematic representation for hydrothermal method (HT).

**Figure** **S4** Schematic representation for photo_ deposition (PCD) method.

**Figure S5** XRD patterns of different metal-loaded TiO_2_ catalysts.

**Figure S6**  XPS for Co/TiO_2_, a) survey, b) O1s, c) Ti2p, and d) Co2p3.

**Figure S7**  XPS for Ni/TiO_2_, a) survey, b) O1s, c) Ti2p, and d) Ni2p3.

**Figure S8** N_2_ adsorption-desorption isotherms for a) Ru_TiO_2_, b) Co_TiO_2_, c) Ni_TiO_2_ nanocatalysts of different metal loading methods, and d) optimized samples relative to TiO_2_(P25).

**Figure S9** Pore size distribution for a) Ru_TiO_2_ , b) Co_TiO_2_ , c) Ni_TiO_2_ of different metal loading methods, and d) optimized samples relative to TiO_2_(P25).

**Figure S10** UV-Vis DRS spectra for a) 0.1 wt.%Ru_TiO_2_, b) 0.3 wt.%Co_TiO_2_ and c) 0.3 wt.%Ni_TiO_2_ with different metal loading methods.

**Figure S11** UV-Vis DRS spectra for a) 0.1 wt.%Ru_TiO_2_ (Imp), b) 0.3 wt.%Co_TiO_2_ (Imp) and c) 0.3 wt.%Ni_TiO_2_ (Imp) of different percent of metal loaded over TiO_2_.

**Figure S12** Initial and cumulative H_2_ production rates over 50 mg samples of a) 0.1wt.%Ru_TiO_2_ (Imp), b) 0.3wt.%Co_TiO_2_ (Imp) and c) 0.3 wt.%Ni_TiO_2_ (Imp) nanocatalysts of different percent of metal loaded over TiO_2_ relative to TiO2(P25), whereas d) shows the relation between initial H_2_ production rates and 50 mg of 0.1wt%Ru_TiO_2_, 0.3 wt.%Co_TiO_2_, and 0.3 wt.%Ni_TiO_2_ nanocomposites of different metal loading methods.

**Figure S13** Cyclic voltammetry (CV) for a) Ru_TiO_2_, b) Ni_TiO_2_, c) Co_TiO_2_ nanocatalysts of different metal loading methods using 0.1M Na_2_SO_4_ electrolyte.
